# Endoscopic treatment of iliopsoas impingement after total hip arthroplasty: a minimum 2-year follow-up and comparison of tenotomy performed at the acetabular rim versus lesser trochanter

**DOI:** 10.1093/jhps/hnab035

**Published:** 2021-05-11

**Authors:** Joaquin Valenzuela, John M O’Donnell

**Affiliations:** 1 Orthopaedic Surgery Department, Clínica Universidad de Los Andes, Plaza 2501, Las Condes, Santiago 7620001, Chile; 2 Orthopaedic Surgery Department, Complejo Asistencial Dr. Sotero del Rio. Concha y Toro 3459, Puente Alto, Santiago. 8207257; 3 Swinburne University of Technology, Hip Arthroscopy Australia, 21 Erin Street, Richmond, Melbourne, VIC 3121, Australia

## Abstract

Iliopsoas impingement is an underdiagnosed cause of groin pain after total hip arthroplasty (THA), being responsible for 4.4% of cases. Non-surgical treatment may be effective in ∼50% of cases. Endoscopic surgery has gained popularity as an option for non-responsive patients because of its non-invasive characteristics, faster recovery and encouraging results. This study compares two different sites of endoscopic psoas tenotomy performed following THA: at the edge of the acetabulum (AR) versus at the lesser trochanter (LT). This is a retrospective review of prospectively collected data from a single-surgeon case series. Thirty-five iliopsoas tenotomy cases which had >24-month follow-up were identified. There were 21 tenotomies at the lesser trochanter. Demographic data, preop and postop pain, mHHS and NAHS scores, strength and patient satisfaction data were collected and analysed. Average age at the time of surgery was 62. Mean follow-up for the LT group was 49.11 months and 42.42 months for the AR group. Pain decreased significantly for both groups (*P* < 0.001). Both mHHS and NAHS showed superiority in the LT group, but this difference did not reach significance (*P* = 0.06). LT patients showed better strength with 71.42% of them having normal strength at latest follow-up, compared with 41.6% in the AR group. There were no complications in either group. Endoscopic tenotomy is a safe and reliable surgical option, giving significant pain relief and good functional outcomes. Tenotomy at the level of the lesser trochanter might be preferable since it shows better outcomes. Larger studies are necessary to achieve statistically significant results.

## INTRODUCTION

Total hip arthroplasty (THA) is one of the most frequently performed and successful procedures in orthopaedics [1]. Its complications, however uncommon, can be devastating for patients and extremely challenging for surgeons. Persistent or new pain after THA can be difficult to address as its causes vary. Pain may be related to infection, component loosening, or wear debris synovitis, but also may be referred from lumbar spine pathology, intra-abdominal and vascular disorders [2]. Iliopsoas tendonitis (IPT) has been reported as a possible cause of pain in up to 24% of patients following hip arthroscopy [3], whereas Iliopsoas impingement is usually an underdiagnosed cause of persistent groin pain in patients following THA, and has been reported to be responsible for up to 4.4% of cases [4–6]. The incidence after revision THA remains unknown.

Patients with iliopsoas impingement usually present complaining of groin pain when going upstairs or walking uphill, rising from a seated position and when getting in or out of a car. It can also be felt like a snapping or ‘clunking’ sensation. Pain can be reproduced on physical examination with resisted hip flexion, especially with the resisted straight-leg-raise, and with stretching of the iliopsoas tendon [7, 8].

Pain resulting from iliopsoas impingement may be related to a prominent and/or malpositioned acetabular component, over-reaming of the anterior wall, long acetabular screws, retained cement and excessive lateralization or lengthening of the extremity during surgery [8–11]. Chalmers *et al.* [7] showed that patients with acetabular prominence < 8 mm had successful results after tenotomy, whereas patients with cup prominence ≥ 8 mm treated with acetabular revision surgery had a higher rate of success compared with tenotomy.

Non-operative treatment includes nonsteroidal anti-inflammatory drugs (NSAIDs), physical therapy and corticosteroid injections, with symptomatic relief of up to 50% of the patients [7, 12, 13]. However, iliopsoas impingement, and the pain that it causes despite non-surgical treatment, can result in major functional disability.

Persistent iliopsoas impingement (IPI) may be treated by an acetabular revision, endoscopic or open psoas tenotomy. Although acetabular revision and psoas tenotomy are both successful alternatives in improving clinical outcomes [13, 14], the latter shows a decreased risk of complications [13, 15]. A recent systematic review published by O’Connell *et al.* [16] comparing open versus arthroscopic tenotomy of the iliopsoas tendon after THA, showed that although both techniques are successful options for the treatment of IPT, the arthroscopic release yields a lower complication rate.

Psoas tenotomy consists in severing the tendon to lengthen the tendon and muscle. This cut can be performed from within the joint cavity at the acetabular rim (AR), where the tendon accounts for 40% of the transverse section of the iliopsoas, or extra-articularly at the level of the lesser trochanter (LT), where the tendon makes up 60% of the iliopsoas. Theoretically, a distal tenotomy could cause greater muscle dysfunction but it could also decrease the risk of recurrence.

AR tenotomy allows the surgeon to further explore the joint if required, but it can potentially increase the risk of anterior instability as reported by Guicherd [17]. On the other hand, extra-articular tenotomy avoids the risk of inadvertent damage of the prosthetic joint and anterior instability, safely gaining access to a greater portion of IPT but with perhaps an increased risk of heterotopic ossification [18].

A recent systematic review evaluated 171 patients who underwent hip arthroscopy after THA, of which 35% were due to IPI, demonstrated that iliopsoas tenotomy in the context of THA is safe, effective and reproducible [19].

To the authors’ knowledge, there are no studies comparing different sites of endoscopic psoas tenotomy performed following THA. We describe minimum 2 year follow-up of patients operated by a single surgeon, aiming to compare pain, strength and functional scores of two anatomical landmarks where the iliopsoas can be released: at the edge of the acetabulum versus at the lesser trochanter.

Our hypothesis in performing the study was that Psoas tenotomy performed at the level of the AR would result in less flexion weakness and higher patient PROM scores and satisfaction.

## MATERIALS AND METHODS

This is a retrospective review of the prospectively collected data of a single surgeon (JOD) case series. Inclusion criteria were patients who had a primary THR, and with minimum two-year follow-up after endoscopic iliopsoas tendon release. Only patients with primary THA were included. Revision THA was an exclusion criterion. Institutional board approval was not required.

Diagnosis was made clinically and confirmed with an ultrasound (US) guided psoas cortisone and local anaesthetic injection. Symptoms were pain and/or snapping during active flexion, and signs were pain reproduction on physical examination with resisted hip flexion and straight leg raise test. AP pelvis and lateral hip X-rays were taken in all cases to exclude prosthetic loosening, but these images were not adequate to accurately assess acetabular component version or anterior wall prominence. MRI and CT scans were not performed. No cups were identified as being oversized or retroverted.

Every patient with suspected IPI underwent initial non-surgical treatment. Non-surgical treatment included oral NSAIDs, supervised physiotherapy for a period of at least 6 weeks, and US guided local anaesthetic and corticosteroid injection (LACI) in all patients. Other causes of pain were excluded. Only patients where non-surgical treatment failed, and where LACI provided marked or complete pain relief for a time, were offered arthroscopic surgical treatment, releasing the iliopsoas tendon either at the acetabular rim or the lesser trochanter level.

Initially, all procedures were performed at the level of the LT, but later the choice of operative approach was made randomly, as both approaches were taught to Fellows. There were no specific indications or contraindications for either procedure, as there has been no definitive study to show which approach is superior.

Patients’ demographic data, surgical approach at the time of THA, onset of symptoms, Visual Analogic Scale (VAS) for pain, and site of iliopsoas tendon release were collected pre-operatively. After surgery information comprised VAS, modified Harris Hip Score (mHHS), Non-Arthritic Hip Score (NAHS), and hip flexion strength [20]. Demographic data and onset of psoas related symptoms after THA data were collected prospectively and reviewed retrospectively.

Latest follow-up was performed telephonically due to the COVID-19 pandemic. Overall satisfaction with the procedure was rated in a simplified manner from 1 to 4, (Unsatisfied, Better than before the surgery, Somewhat Satisfied, Very Satisfied), given the telephonical nature of the follow-up.

The two groups of patients were compared in terms of their characteristics before and after their procedures. The two groups were then compared in terms of outcomes and change scores. Use was made of 2-sided *t*-tests in the case of metric variables with 1000 bootstrap samples used to calculate *P*-values and 2-sided Exact Fisher tests in the case of categorical variables. A follow-up paired *t* test was used to assess the significance of pain reduction for both groups separately. In all cases a 5% significance level was assumed with the analysis conducted using IBM SPSS Statistics v27.

Two different surgical techniques were used. For tenotomies at the level of the acetabular rim, patients were positioned in a lateral decubitus position, without traction. Anterolateral and mid-anterior portals were used, giving access to the central compartment and the psoas notch. Inspection of the joint was performed, and this was followed by anterior capsulotomy to access the Psoas tendon. For tenotomies at the level of the LT, patients were supine, with a bolster under the contralateral hip for facilitating external rotation of the leg undergoing the procedure. Under fluoroscopy, portals were created using cannulated needles, flexible nitinol guides and dilators, as in a standard hip arthroscopy. Instruments were introduced aiming ∼2 cm proximal to the lesser trochanter. In all cases, complete iliopsoas tenotomy was performed, including any additional tendons, which have been reported to be present in up to 70% of patients [21] and retraction of the proximal tendon(s) was confirmed visually.

Physiotherapy for psoas tenotomy surgery starts 2 weeks prior to surgery, with education on posture specifically to keep hip flexion under 90° while sitting in order to minimize irritation of the anterior capsule, and activities of daily living. Gait aid will be measured and practiced including ascending and descending stairs. Following surgery, icing 20 min every 2 h for 5 days is utilized to minimize swelling and soreness. Hip flexion/straight leg raising, and stretching exercises are avoided for the initial 6 weeks.

Post-operatively, patients used a Compressive Cryotherapy device [22], and a standard analgesic and rehabilitation protocol. Every patient was discharged the day after the surgery was performed and received heterotopic bone prophylaxis with NSAIDs (Meloxicam 15 mg orally, daily) for 14 days [23].

## RESULTS

Thirty-five patients with a minimum 24-month follow-up were included, with no loss to follow-up. Mean age at the time of surgery was 62 years (range: 40–84, SD ± 10.33), with 20 females (57.14%). Mean Body Mass Index (BMI) was 28.74 (range: 20.3–41.1, SD ± 4.94). Surgery was performed on 23 right iliopsoas and 12 left. Mean follow-up was 49.11 months (SD 20.46) and 42.42 months (SD 12.25) for the LT group and AR group, respectively (*P* = 0.268).

Data regarding time of onset of symptoms after THA were available for 33 out of 35 (94.28%). Five patients (15.15%) reported symptoms which commenced immediately after surgery; nineteen patients (57.57%) had symptoms which commenced between 1 and 12 months post-operatively; and nine patients (27.27%) symptoms commenced more than one year after surgery.

Regarding THA approach, we collected information of 33 out of 35 patients (94.28%). Anterior and Posterior approaches were the most frequently reported surgical approaches, with 19 (57.5%) and 11 (33.3%) cases, respectively. Anterolateral approach accounted for only 3 (9.0%) cases in this series.

Table I shows a comparison of the procedure groups pre-surgery for the categorical variables while Table II considers the metric variables. No significant differences were found between the groups, suggesting that the groups were well matched.

**Table I.** Comparison of procedure groups for categorical variables

		Sample size by group(%)		Fisher exact (*P*-value)
		LT	AR	
Gender	Female	12 (57.1)	8 (57.1)	1.000
	Male	9 (42.9)	6 (42.9)	—
Surgery side	Left	6 (28.6)	6 (42.9)	0.477
	Right	15 (71.4)	8 (57.1)	—
Onset after THR	At most 12 months	15 (75.0)	9 (69.2)	0.509
	More than 12 months	5 (25.0)	4 (30.8)	—
Surgery approach	Anterior	12 (57.1)	7 (50.0)	0.935
	Anterolateral	2 (9.5)	1 (7.1)	—
	No information	1 (4.8)	1 (7.1)	—
	Posterior	6 (28.6)	5 (35.7)	—

**Table II.** Comparison of procedure groups for pre-surgery metric variables

	Mean (std dev)		Effect size Cohen’s *d*	Bootstrap *P*-value
	LT	AR		
Follow-up months	49.11 (20.46)	42.42 (12.25)	0.376	0.268
Age at surgery (years)	63.95 (10.66)	59.92 (8.34)	0.409	0.265
Height (mm)	168.37 (9.40)	168.67 (9.31)	0.032	0.920
Weight (kg)	83.89 (18.89)	76.33 (7.48)	0.485	0.134
BMI	29.49 (5.54)	26.98 (3.34)	0.520	0.116
Time to onset	0.263 (0.452)	0.33 (0.49)	0.150	0.686
VAS preop	5.32 (1.06)	5.75 (1.29)	0.378	0.314

Arthroscopic iliopsoas tenotomy was performed at the lesser trochanter (LT) in 21 patients and 14 patients underwent tenotomy at the level of the acetabular rim (AR). Post-operatively, all patients were discharged within 24 h and allowed to fully weight bear as tolerated. Physiotherapy was advised for every patient. There were neither early nor late surgical complications and specifically, there were no infections, dislocations or heterotopic calcification, and there were no readmissions to hospital.

Pain was measured using the Visual Analogue Scale (VAS) pre-operatively and post-operatively. Mean VAS score pre-operatively was 5.32 (SD 1.06) for the LT group and 5.75 (SD 1.29) in AR patients. Post-operative pain decreased for every patient. Mean pre-operative pain score improved significantly from 5.32–1.75 in the LT group (*P*<.001), whereas for the AR group it improved significantly from a mean of 5.75–2.62 (*P*<.001). Ten patients reported 0 pain at latest follow-up, six for the LT group and four in the AR group.

mHHS was measured at the latest follow-up for both groups. Mean mHHS in the LT group was 88.98 (SD10.29), and 81.05 (SD 12.44) for the AR group showing a nearly significant difference between the procedures (*P* = 0.068) and a large effect size (Table III).

**Table III.** Comparison of the current outcome measures for the two groups at final follow-up

	Mean (std dev)		Effect size Cohen’s *d*	Bootstrap *P*-value
	LT	AR		
mHHS	88.98 (10.29)	81.05 (12.44)	0.709	0.068
NAHS	85.99 (11.11)	78.85 (10.10)	0.666	0.067
VAS	1.75 (1.77)	2.62 (2.22)	0.442	0.224
Change in VAS	3.60 (1.54)	3.08 (2.10)	0.295	0.415
Satisfaction	3.19 (0.93)	2.69 (1.18)	0.483	0.180

mHHS, modified Harris Hip Score; NAHS, Non-Arthritic Hip Score; VAS, Visual Analogue Scale.

The NAHS was also measured at the latest follow-up in both groups. The mean NAHS for the LT group was 85 (11.11), and for the AR group the mean was 78.85 (SD 10.1) also showing a nearly significant difference between the procedures (*P* = 0.067) and a moderate to large effect size in Table III.

Strength was measured using a 0–5 score [20]. All patients had significant weakness pre-operatively, probably mainly due to pain. Post-operatively, data were obtained from 33 out of 35 patients, of which 20 (60.6%) reported normal strength, eight (24.24%) had 04 out of 05 scores; and five patients had 03 out of 05 (15.15%). If groups are compared, 15 out of 21 patients (71.42%) have normal strength at latest follow-up in the LT group, compared with 5 out of 12 (41.6%) in the AR group. However, no significant difference was observed between the LT and AR procedures in regard to strength (Fisher Exact test *P* values = 0.083).

Overall satisfaction, rated from 1 to 4, with the procedure, showed a high percentage of satisfied patients (70.58%), although four patients reported that they were unsatisfied (11.1%). The LT group had a higher satisfaction rate at 76.19%, compared with 53.84% in the AR group, which actually contained 3 of the 4 very unsatisfied patients. As shown in Table III there was no significant difference between the mean satisfaction scores for the LT and AR groups.

## DISCUSSION

Treatment of recalcitrant IPT by endoscopic tendon lengthening resulted in improved levels of pain and function, whether performed at the AR or LT. Tenotomy performed at the level of AR did not result in superior outcomes.

Accurate diagnosis and treatment of groin pain after THR remain a challenge. Iliopsoas impingement causing bursitis/tendonitis is a common source of groin pain after THA, being responsible for 4.4% of the cases of groin pain [4–6]. The reported success of conservative treatment for Iliopsoas tendinopathy has varied very widely. Conservative treatment, requiring up to three fluoroscopically guided injections, has been reported, leading to improved symptoms in as many as 50–78% of the patients in some series [5, 6, 12]. However, other authors have reported failure in 100% of cases [13] treated conservatively. The cause of this disparity is not clear.

Our study shows that both surgical techniques analysed are reliable in terms of pain relief, functional results, strength and patient satisfaction. However, because of the rapid growth in arthroscopic surgical techniques, often surgeons have been left following trends based on limited, or even anecdotal, evidence, instead of solid, well designed studies [24], and with no clear algorithm to follow. For that reason, in our study there was no algorithm guiding the surgical choice for each patient. Rather, all patients were initially treated by tenotomy at the LT. Because of suggestions by other surgeons that tenotomy at the acetabular rim may, in theory, be associated with less post-operative weakness, the senior author adopted this technique also. This post-operative weakness theory is based on the fact that at the AR the IPT comprises just 40% of the iliopsoas, compared with 60% at the lesser trochanter [25]. For that reason, the distal tenotomy could potentially cause greater muscle function loss but for the same reason it should decrease the risk of recurrence.

Our study confirms findings from Guicherd *et al.* [17], showing good results regarding muscle strength, achieving normal strength in 20/33 patients (60.6%) and 4 out of 5 score in an additional 24.24% at latest follow-up. Nevertheless, patients in the LT group consistently reported normal (5/5) or almost normal (4/5) strength in 95.23% of the cases, compared with 66.6% in the AR group. However, the difference between groups was not significant (*P* = 0.083). These findings appear to contradict the theoretical risk of loss of hip flexion strength secondary to the disruption of the muscle-tendon complex [24]. This might be due to the fact the iliopsoas comprises multiple bundles, such as the iliocapsularis, inserted on the capsule and metaphysis, as well as the infratrochanteric bundle [25, 26].

Pain, as measured by the VAS, showed significant improvement in both groups, from an average 5.32 in the LT group and 5.75 in the AR group pre-operatively, to 1.75 and 2.62, respectively, in their latest follow-up (*P* < 0.001). However, there was no significant difference between groups (*P*** **= 0.415).

Regarding hip arthroplasty approach, 19 (57.57%) of the studied population had an anterior approach, compared with 11 (33.33%) posterior approaches. Due to the fact that the senior author uses an anterior approach for his THA patients and most patients come from his practice, we can’t draw any conclusions with this data. Nonetheless, it might be an interesting topic to analyse on further studies.

Functional scores measured at latest follow-up, mHHS and NAHS, were higher for the LT group showing a nearly significant difference between the procedures but with a large effect size for the mHHS and a moderate to large effect size for the NAHS. In addition, the mHHS, effectively correlates with patient satisfaction [27], 89.47% patients in the LT group had a score above the patient accepted symptomatic state (PASS) as reported by Chahal *et al.* [28], as opposed to just 61.53% in the AR group.

In addition to these reported advantages of the LT tenotomy over the AR tenotomy, for the latter, there is an additional risk of damaging the bearings of the THA inadvertently with the arthroscopic instruments, as well as some small risk of bacterial contamination of the prosthetic joint, and a risk of joint instability due to the capsulotomy at the level of the iliofemoral ligament [17, 25]. The risk is greater in the case of excessive femoral anteversion [29]. In our series, no dislocation, infection or damaged bearings were found.

To our knowledge this is the largest single-surgeon series of arthroscopic/endoscopic tenotomy treatment for iliopsoas impingement, and the first one to compare the results of tenotomy performed at the lesser trochanter, and at the acetabular rim. Every patient followed the same standardized diagnosis workup and pre-operative treatment, as well as the same surgical technique (in each group) and post-operative care [22].

This study has several weaknesses. Data were collected retrospectively, and as a result, some data points are missing. Many mHHS and NAHS pre-operative values were not collected. However, patients consistently had scores above the PASS, with better results reported for the LT group. Specific radiographical assessment of the degree of acetabular prominence was not performed.

## CONCLUSION

This study supports arthroscopic/endoscopic surgical management for patients with iliopsoas impingement after THA, which is not responsive to non-surgical treatment. Both tenotomy techniques led to improved symptoms in a high percentage of patients and had no early, or late, complications. However, tenotomy at the LT consistently showed less post-operative pain, better strength and higher functional scores in comparison with the AR group, although these results did not reach statistical significance. Although these results suggest that both are reliable surgical options, with a trend for better outcomes for the LT group, a larger, prospective trial is required in order to definitively determine which technique is superior.
